# Performance Evaluation of the Gradient Diffusion Strip Method and Disk Diffusion Method for Ceftazidime–Avibactam Against *Enterobacterales* and *Pseudomonas aeruginosa*: A Dual-Center Study

**DOI:** 10.3389/fmicb.2021.710526

**Published:** 2021-09-16

**Authors:** Jingjia Zhang, Gang Li, Ge Zhang, Wei Kang, Simeng Duan, Tong Wang, Jin Li, Zhiru Huangfu, Qiwen Yang, Yingchun Xu, Wei Jia, Hongli Sun

**Affiliations:** ^1^Department of Clinical Laboratory, Peking Union Medical College Hospital, Peking Union Medical College, Chinese Academy of Medical Sciences, Beijing, China; ^2^Medical Experimental Center, General Hospital of Ningxia Medical University, Yinchuan, China

**Keywords:** ceftazidime-avibactam, gradient diffusion strips method, broth microdilution, disk diffusion, evaluation

## Abstract

**Objectives:** Ceftazidime–avibactam is a novel synthetic beta-lactam + beta-lactamase inhibitor combination. We evaluated the performance of the gradient diffusion strip method and the disk diffusion method for the determination of ceftazidime–avibactam against *Enterobacterales* and *Pseudomonas aeruginosa.*

**Methods:** Antimicrobial susceptibility testing of 302 clinical *Enterobacterales* and *Pseudomonas aeruginosa* isolates from two centers were conducted by broth microdilution (BMD), gradient diffusion strip method, and disk diffusion method for ceftazidime–avibactam. Using BMD as a gold standard, essential agreement (EA), categorical agreement (CA), major error (ME), and very major error (VME) were determined according to CLSI guidelines. CA and EA rate > 90%, ME rate < 3%, and VME rate < 1.5% were considered as acceptable criteria. Polymerase chain reaction and Sanger sequencing were performed to determine the carbapenem resistance genes of all 302 isolates.

**Results:** A total of 302 strains were enrolled, among which 182 strains were from center 1 and 120 strains were from center 2. A percentage of 18.21% (55/302) of the enrolled isolates were resistant to ceftazidime–avibactam. The CA rates of the gradient diffusion strip method for *Enterobacterales* and *P. aeruginosa* were 100% and 98.65% (73/74), respectively, and the EA rates were 97.37% (222/228) and 98.65% (73/74), respectively. The CA rates of the disk diffusion method for *Enterobacterales* and *P. aeruginosa* were 100% and 95.95% (71/74), respectively. No VMEs were found by using the gradient diffusion strip method, while the ME rate was 0.40% (1/247). No MEs were found by using the disk diffusion method, but the VME rate was 5.45% (3/55). Therefore, all the parameters of the gradient diffusion strip method were in line with acceptable criteria. For 31 *bla*_*KPC*_, 33 *bla*_*NDM*_, 7 *bla*_*IMP*_, and 2 *bla*_*VIM*_ positive isolates, both CA and EA rates were 100%; no MEs or VMEs were detected by either method. For 15 carbapenemase-non-producing resistant isolates, the CA and EA rates of the gradient diffusion strips method were 100%. Whereas the CA rate of the disk diffusion method was 80.00% (12/15), the VME rate was 20.00% (3/15).

**Conclusion:** The gradient diffusion strip method can meet the needs of clinical microbiological laboratories for testing the susceptibility of ceftazidime–avibactam drugs. However, the VME rate > 1.5% (5.45%) by the disk diffusion method. By comparison, the performance of the gradient diffusion strip method was better than that of the disk diffusion method.

## Introduction

The increase in the isolation rate of multidrug-resistant Gram-negative bacilli has become a public health issue of global concern (Tangden and Giske, 2015). Isolates usually develop resistance to β-lactam antibiotics, which greatly limits the treatment options for severe bacterial infections (Bush, 2010). The prevalence of pathogenic bacteria producing extended-spectrum β-lactamase (ESBL) remains high, leading to an increase in the use and dependence of carbapenem antimicrobials. Therefore, the emergence and spread of carbapenemase-producing pathogens (including carbapenem-resistant *Acinetobacter baumannii*, *Pseudomonas aeruginosa*, and *Enterobacterales*) have attracted more and more attention. New antibacterial agents have also become an urgent clinical need (Tangden and Giske, 2015; Kazmierczak et al., 2018).

Ceftazidime–avibactam is an intravenously administered combination of the third-generation cephalosporin, ceftazidime, and the novel, non-β-lactam β-lactamase inhibitor avibactam (Shirley, 2018). Traditional β-lactamase inhibitors (clavulanic acid, tazobactam, and sulbactam) lack activity on many important β-lactamases, so the first-generation β-lactam/β-lactamase enzyme inhibitor combinations are usually ineffective against multidrug-resistant pathogens (Bebrone et al., 2010). Avibactam is a non-β-lactam β-lactamase inhibitor that inhibits the activity of Ambler class A β-lactamases, including ESBLs and KPC carbapenemases; AmpC cephalosporinases (Ambler class C β-lactamases); and some Ambler class D β-lactamases (Ehmann et al., 2012; Shields et al., 2017; Karlowsky et al., 2018). Ceftazidime, a third-generation cephalosporin, has broad-spectrum activity against gram-negative bacilli, including *P. aeruginosa*. It in combination with avibactam has proven potent *in vitro* activity against KPC-producing clinical isolates of *Enterobacterales* (Nichols et al., 2016). It does not inhibit class B metallo-β-lactamases that have a catalytic zinc atom in the active site (Hackel et al., 2016; Kazmierczak et al., 2018).

In this study, we aimed to evaluate two commonly used methods for clinical detection of the antimicrobial susceptibility of ceftazidime–avibactam. Therefore, we evaluated the performance of the gradient diffusion strip method and the disk diffusion method to detect the susceptibility of *Enterobacterales* and *P. aeruginosa*. Currently, no other commercial products are available for testing in China. Wang et al. (2020) did a similar study in 2020, but it was a single-center study, and the isolates came from the same hospital. Our study was a dual-center study, the collected isolates had a wider source, and the data were more universal.

## Materials and Methods

### Isolates

This study is a dual-center study. We collected a total of 302 isolates from Peking Union Medical College Hospital (center 1) and People’s Hospital of Ningxia Hui Autonomous Region (center 2) from 2018 to 2020. Among them, 182 isolates were from center 1, and 120 isolates were from center 2 (Supplementary Table 1). This study plans to collect 30% of isolates resistant to meropenem. All isolates were identified using MALDI-TOF MS (Vitek MS, bioMérieux, France). All duplicate isolates (the same genus and species from the same patient) were excluded. Isolates were stored at −80°C in a cryotube with 20% (w/v) skimmed milk until subcultured on Blood Agar Plate (Oxoid, Basingstoke, United Kindom). *Escherichia coli* ATCC 25922, *Klebsiella pneumoniae* ATCC 700603, *P. aeruginosa* ATCC 27853, and *E. coli* ATCC 35218 were used as quality control strains.

### Antimicrobial Susceptibility Testing

The gradient diffusion method was evaluated using the E-test strips (bioMérieux, Marcy-l’Etoile, France) and utilized according to the manufacturer’s instructions. The concentration gradient of ceftazidime ranged from 0.016 to 256 μg/ml with avibactam at a fixed concentration of 4 μg/ml. The E-test strips can provide results between conventional doubling dilutions (e.g., 0.19, 0.38, 0.75, 1.5, 3, 6, 12). For minimum inhibitory concentrations (MICs) between the standard two-fold dilution values, the result was rounded to the next higher standard value (e.g., when the MIC was 0.75/4 mg/l obtained by the gradient diffusion strip method, it should be normalized to 1/4 mg/l).

Broth microdilution (BMD) MICs were determined according to the CLSI M100S28 document (CLSI, 2020). The concentration gradient of ceftazidime–avibactam in the BMD panel was 0.016/4 to 256/4 μg/ml. The clinical breakpoints of these two methods for susceptibility and resistance were ≤8/4 and ≥ 16/4 mg/l (CLSI, 2020).

The ceftazidime–avibactam disks (30/20 μg) were obtained from Oxoid (Hampshire, United Kingdom). The disk diffusion test was carried out according to the CLSI M2 document (CLSI, 2018). The diameter of the inhibition zone was measured with a Vernier caliper. The clinical breakpoints of the disk diffusion method for susceptibility and resistance were ≥21 mm and ≤20 mm, respectively (CLSI, 2020).

All 302 isolates were tested for antimicrobial susceptibility by the three methods in the center 1 laboratory. The three methods used the same inoculum of 0.5 McFarland.

### Agreement Analysis

Using BMD as a gold stand, categorical agreement (CA), essential agreement (EA), major error (ME), and very major error (VME) were calculated according to the CLSI M52 document (CLSI, 2015). Results were considered CA when isolates had the same susceptible, intermediate, susceptible dose-dependent, and resistant category as the BMD method category result. Results were considered EA when the MIC was obtained with the gradient diffusion strip method that was within one doubling dilution step (two-fold serial) from the MIC value established with the BMD method. Results were considered ME when the BMD method result was susceptible and the gradient diffusion strip method or disk diffusion was resistant. Results were considered VME when the BMD method result was resistant and the result from the gradient diffusion strip method or disk diffusion was susceptible. CA and EA > 90%, ME < 3%, and VME < 1.5% were considered as acceptable criteria.

The Pearson correlation coefficients (*p*-value) were calculated using SPSS ver. 26.0. A linear regression curve analysis was performed using GraphPad Prism ver. 8, and the R square value was calculated.

### Screening of Carbapenemase Genes

Polymerase chain reaction (PCR) and Sanger sequencing were used to screen out carbapenemase genes including *bla*_*KPC*_, *bla*_*NDM*_, *bla*_*VIM*_, *bla*_*IMP*_, and *bla*_*OXA–*__48_. The oligonucleotide sequences of the primers are listed in Supplementary Table 2 (Yigit et al., 2001; Shibata et al., 2003; Boutal et al., 2018). The PCR products were sequenced and analyzed using BLAST.^1^

## Results

### Isolate Information

**FIGURE 1:**
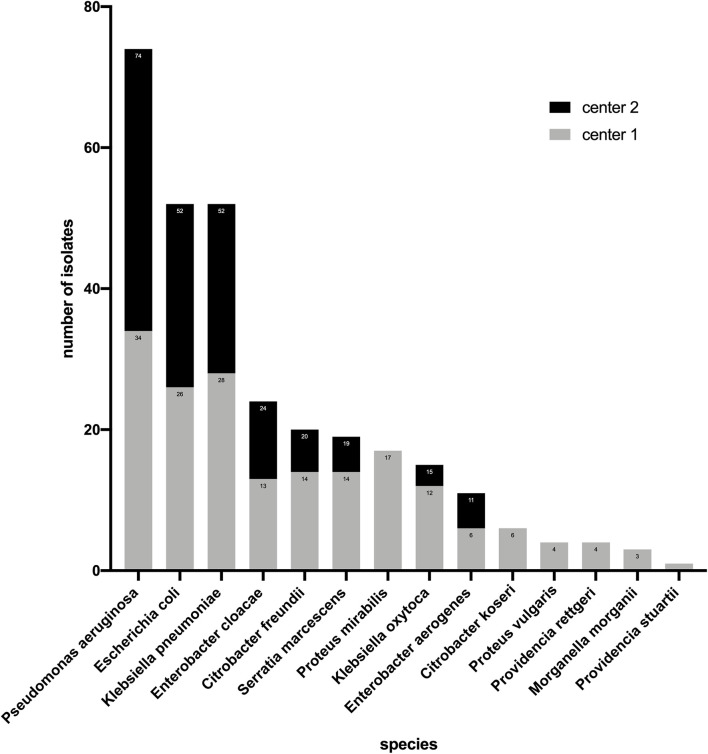
Distribution of isolates tested in center 1 and center 2 (*n* = 302).

In this study, 74 strains of *P. aeruginosa* and 228 strains of *Enterobacterales* were investigated. Among the *Enterobacterales*, *E. coli* (*n* = 52) and *Klebsiella pneumoniae* (*n* = 52) accounted for the highest proportion followed by *Enterobacter cloacae* (*n* = 24) and *Citrobacter freundii* (*n* = 20), *Morganella morganii* (*n* = 3), and *Providencia stuartii* (*n* = 1) had the smallest proportion (Figure 1). For *Enterobacterales*, the three methods had the same number (*n* = 41) of strains resistant to ceftazidime–avibactam. However, this number was different for *P. aeruginosa*, BMD, and the gradient diffusion strip method which detected 14 and 15 resistant isolates, respectively, while disk diffusion only detected 11 (Table 1).

**TABLE 1:** Performance of the gradient diffusion strip method and disk diffusion compared with BMD for *Enterobacterales* and *P. aeruginosa*.

Organism	No.	No. of resistant isolates N (%)			ETEST vs. BMD N (%)				Disk diffusion vs. BMD N (%)		
		BMD	ETEST	Disk diffusion	CA	EA	ME	VME	CA	ME	VME
***Enterobacterales***											
Center 1	148	25(16.89)	25 (16.89)	25 (16.89)	148 (100)	145 (97.97)	0 (0)	0 (0)	148 (100)	0 (0)	0 (0)
Center 2	80	16 (20.00)	16 (20.00)	16 (20.00)	80 (100)	77 (96.25)	0 (0)	0 (0)	80 (100)	0 (0)	0 (0)
Total in *Enterobacterales*	228	41 (17.98)	41 (17.98)	41 (17.98)	228 (100)	222 (97.37)	0 (0)	0 (0)	228 (100)	0 (0)	0 (0)
***P. aeruginosa***											
Center 1	34	11 (32.35)	12 (35.29)	8 (23.53)	33 (97.06)	33 (97.06)	1 (4.35)	0 (0)	31 (91.18)	0 (0)	3 (27.27)
Center 2	40	3 (7.50)	3 (7.50)	3 (7.50)	40 (100)	40 (100)	0 (0)	0 (0)	40 (100)	0 (0)	0 (0)
Total in *P. aeruginosa*	74	14 (18.92)	15 (20.27)	11 (14.86)	73 (98.65)	73 (98.65)	1 (1.67)	0 (0)	71 (95.95)	0 (0)	3 (21.43)
**Total**											
Center 1	182	36 (19.78)	37 (20.33)	33 (18.13)	181 (99.45)	178 (97.8)	1 (0.68)	0 (0)	178 (97.8)	0 (0)	3 (8.33)
Center 2	120	19 (15.83)	19 (15.83)	19 (15.83)	120 (100)	117 (97.5)	0 (0)	0 (0)	120 (100)	0 (0)	0 (0)
Total in all tested isolates	302	55 (18.21)	56 (18.54)	52 (17.22)	301 (99.67)	295 (97.68)	1 (0.40)	0 (0)	298 (98.68)	0 (0)	3 (5.45)

*BMD, broth microdilution; CA, categorical agreement; EA, essential agreement; ME, major error; VME, very major error.*

### Genotype Determination

Polymerase chain reaction (PCR) and Sanger sequencing were used to screen carbapenem-resistant genes of all 302 isolates, which are shown in Table 2. A total of 71 strains were detected carrying four types of resistance genes: *bla*_*KPC–*__2_, *bla*_*NDM*_, *bla*_*IMP*_, and *bla*_*VIM*_. NDM and KPC accounted for similar proportions being 45.07% (32/71) and 43.66% (31/71), respectively. Among them, there was *bla*_*KPC*_ coupled with *bla*_*NDM*_ positive. The percentage of IMP and VIM was 9.86 (7/71) and 2.82 (2/71), respectively. This study failed to collect *bla*_*OXA–*__48_-positive isolates.

**TABLE 2:** The gene type of 302 isolates.

Organism	The number of isolates with different carbapenemase resistance mechanisms											
	KPC		NDM		IMP		VIM		OXA-48		Not detected	
	Center 1	Center 2	Center 1	Center 2	Center 1	Center 2	Center 1	Center 2	Center 1	Center 2	Center 1	Center 2
*Citrobacter freundii*			1			1					13	5
*Citrobacter koseri*											6	
*Enterobacter aerogenes*												5
*Enterobacter cloacae*	2		7	2	1	2					3	7
*Escherichia coli*	2		9	4							15	21
*Klebsiella aerogenes*											6	
*Klebsiella oxytoca*	2		1		2						8	3
*Klebsiella pneumoniae*	21		2	6							5	18
*Morganella morganii*											3	
*Proteus mirabilis*											17	
*Proteus vulgaris*											4	
*Providencia rettgeri*											4	
*Providencia stuartii*											1	
*Pseudomonas aeruginosa*						1					32	39
*Serratia marcescens*	4						2				10	5
Total	31		32		7		2		0		230	

*KPC, *Klebsiella pneumoniae* carbapenemase; NDM, New Delhi metallo-beta-lactamase; VIM, Verona integron-borne metallo-beta-lactamase; IMP, imipenemase; OXA, oxacillinase.*

Of the 71 isolates, center 1 accounted for a larger proportion of 77.46% (55/71) and center 2 accounted for 22.53% (16/71). As shown in Table 2, the 16 isolates of center 2 were all class B metallo-β-lactamases (12 NDM and 4 IMP). Center 2 did not collect *bla*_*KPC*_-positive isolates due to region specificity.

### Performance of Gradient Diffusion Strips Method

**FIGURE 2:**
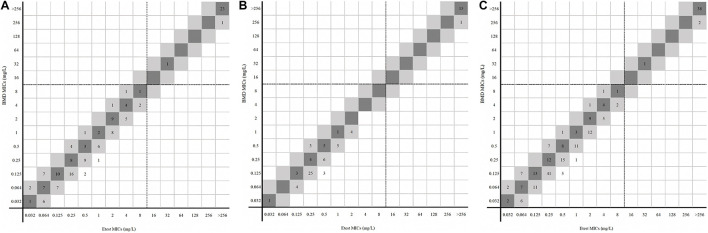
MICs of *Enterobacterales* determined by gradient diffusion strips method and BMD. (**A**, center 1), (**B**, center 2), and (**C**, total) were the results of gradient diffusion strips method versus BMD. Dark gray expresses identical MIC, and light gray indicates twofold difference between the BMD and gradient diffusion strips method MICs. Dotted lines show the clinical breakpoints for each antibiotic.

For 228 *Enterobacterales* isolates, no MEs or VMEs were detected. The CA rate was 100%. The EA rate was 97.37%, the center 1 and center 2 rates being 97.97% and 96.25%, respectively (Table 1). Among the 41 resistant isolates detected by the two methods, only one had an MIC between 16/4 and 128/4 mg/l (32/4 mg/l), and the MICs of others were all greater than 128/4 mg/l (Figure 2).

**FIGURE 3:**
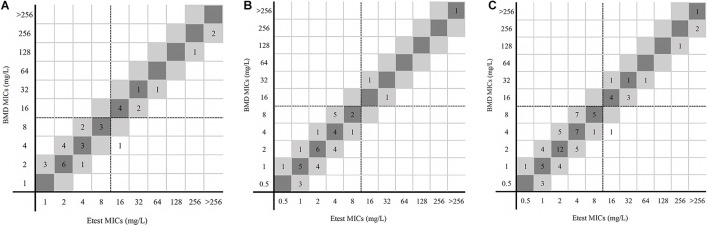
MICs of *P. aeruginosa* determined by gradient diffusion strips method and BMD. (**A**, center 1), (**B**, center 2), and (**C**, total) were the results of gradient diffusion strips method versus BMD. Dark gray expresses identical MIC, and light gray indicates twofold difference between the BMD and gradient diffusion strips method MICs. Dotted lines show the clinical breakpoints for each antibiotic.

For 74 *P. aeruginosa*, no VME was detected. Only one isolate in center 1 was tested for resistant by the gradient diffusion strip method but susceptible by BMD. The ME rate was 1.67% (1/60). The CA and EA rates of 74 *P. aeruginosa* were both 98.65%. The CA and EA rates of center 1 were both 97.06% and for center 2 100% (Table 1). Figure 3 shows the distribution of *P. aeruginosa* MIC determined by the gradient diffusion strip method and BMD.

### Performance of Disk Diffusion Method

**FIGURE 4:**
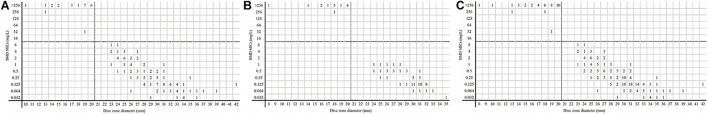
MICs and disk zone diameters of *Enterobacterales* determined by BMD and the disk diffusion method. (**A**, center 1), (**B**, center 2), and (**C**, total) were the results of the disk diffusion method versus BMD. Dotted lines show the clinical breakpoints for each antibiotic.

Figure 4 shows the results of disk diffusion and BMD for 228 *Enterobacterales* isolates. The CA rate was 100%. In the 41 resistant isolates detected by the disk diffusion, none had an inhibition zone diameter of 6 mm. More than half of the resistant isolates (26/41) fell in the range 18–20 mm.

**FIGURE 5:**
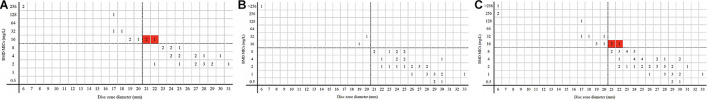
MICs and disk zone diameters of *P. aeruginosa* determined by BMD and the disk diffusion method. (**A**, center 1), (**B**, center 2), and (**C**, total) were the results of the disk diffusion method versus BMD. Dotted lines show the clinical breakpoints for each antibiotic. Red cubes indicated VME.

For 74 *P. aeruginosa*, no ME was detected, while three isolates in center 1 were tested as susceptible by disk diffusion but resistant by BMD. The VME rate was 21.43% (3/14). The CA rate was 95.95%, center 1 and center 2 being 91.18% and 100%, respectively. Three isolates had 6-mm inhibition zone diameters in 11 resistant isolates detected by disk diffusion; others (8/11) were in the range 17–20 mm (Figure 5).

### Performance of the Gradient Diffusion Strip Method vs. the Disk Diffusion Method

The CA rates of *Enterobacterales* obtained by the two methods were both 100%; those obtained by the gradient diffusion strips method were greater than the disk diffusion method (98.65% vs. 95.95%) for *P. aeruginosa*. For *P. aeruginosa*, the VME rate tested by the gradient diffusion strip method and disk diffusion method were 0% and 21.43% (3/14), respectively. The ME rate obtained by disk diffusion was 0% and 1.67% (1/60) detected by the gradient diffusion strip method.

**FIGURE 6:**
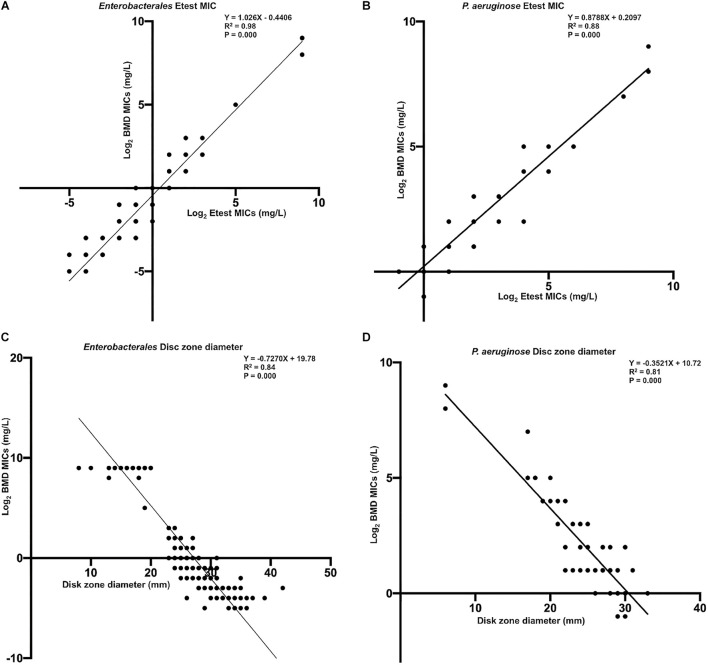
The linear regression curve between the MICs determined by gradient diffusion strips method, disk diffusion, and BMD. (**A**, *Enterobacterales*) and (**B**, *P. aeruginosa*) were curves between gradient diffusion strips method and BMD; (**C**, *Enterobacterales*) and (**D**, *P. aeruginosa*) were curves between the disk diffusion method and BMD.

Figures 6A,B show the linear regression curves between the MICs determined by the gradient diffusion strip method and BMD. The R squared values for *Enterobacterales* and *P. aeruginosa* were 0.98 and 0.88, respectively. Figures 6C,D show the linear regression curves between the disk zone diameter and the MIC of BMD. The R squared values were 0.82 and 0.74, and the *p*-values were all <0.001. It can be seen that the goodness of fit between the results of the gradient diffusion strip method and BMD was better than that between disk diffusion and BMD, and the fit of *Enterobacterales* was superior to that of *P. aeruginosa*.

### Performance Evaluation Against Isolates With Different Carbapenemase Genotypes

A total of 55 resistant isolates were detected by BMD (Table 1) including 31 resistant *bla*_*NDM*_-positive, 7 *bla*_*IMP*_-positive, and 2 *bla*_*VIM*_-positive isolates; 15 isolates were not found to carry any genes (Table 2). The 40 isolates carrying antibiotic-resistant genes all showed high-level resistance (MICs > 256/4 mg/l) to ceftazidime–avibactam. In addition, all of the 30 isolates that only carried KPC were susceptible to ceftazidime–avibactam, but *Klebsiella oxytoca bla*_*KPC*_ coupled with *bla*_*NDM*_ positive was resistant.

For 14 resistant *P. aeruginosa* isolates tested by BMD, 11 were carbapenemase-non-producing. With the exception of one isolate with MIC of 128/4 mg/l, all other isolates showed low-level resistance (MIC was 16/4 or 32/4 mg/l) and were very close to the clinical breakpoint.

Table 3 shows the rates of CA, EA, ME, and VME for different carbapenemase resistance mechanisms. The CA and EA rates measured by the gradient diffusion strips method were all 100%. The CA rate of carbapenemase-non-producing isolates detected by disk diffusion was 80.00% (12/15), with all three isolates tested as susceptible by the disk diffusion but were resistant by BMD in this study; the VME rate was 20.00% (3/15).

**TABLE 3:** Performance of the gradient diffusion strip method and disk diffusion compared with different carbapenemase resistance mechanisms.

Gene	ETEST vs. BMD N (%)				Disk diffusion vs. BMD N (%)		
	CA	EA	ME^*a*^	VME	CA	ME^*a*^	VME
*KPC*(31)	31 (100)	31 (100)	0 (0)	0 (0)	31 (100)	0 (0)	0 (0)
*NDM*(33)	32 (100)	32 (100)	0 (0)	0 (0)	33 (100)	0 (0)	0 (0)
*IMP*(7)	7 (100)	7 (100)		0 (0)	7 (100)		0 (0)
*VIM*(2)	2 (100)	2 (100)		0 (0)	2 (100)		0 (0)
*Notdetected*(15)	15 (100)	15 (100)		0 (0)	12 (80.00)		3 (20.00)

*^*a*^In addition to KPC and NDM, several other types were resistant to ceftazidime–avibactam, so the ME rate cannot be calculated. BMD, broth microdilution; CA, categorical agreement; EA, essential agreement; ME, major error; VME, very major error; KPC, Klebsiella pneumoniae carbapenemase; NDM, New Delhi metallo-beta-lactamase; VIM, Verona integron-borne metallo-beta-lactamase; IMP, imipenemase; OXA, oxacillinase.*

## Discussion

In China, carbapenem antibiotics are considered to be the most effective antimicrobial drugs for the treatment of infections caused by multidrug resistance (MDR) and extensive drug resistance (XDR) Gram-negative bacilli (Yin et al., 2019). However, the numbers of carbapenem-resistant *Enterobacterales* (CRE) have continued to increase in recent years. The CHINET Antimicrobial Surveillance Network in 2018 showed that more than 25% of *K. pneumoniae* isolates were resistant to imipenem and meropenem, a near 10 times increase since 2005 (Yang et al., 2020). Ceftazidime–avibactam is a more effective antibiotic used to treat carbapenem-resistant strains. Previous studies had reported that ceftazidime–avibactam had a better effect on Gram-negative bacterial infections (Shields et al., 2017; Santevecchi et al., 2018; Shirley, 2018). However, the laboratory testing methods to investigate the bacterial susceptibility to this drug still needed to be explored.

This was the first dual-center study to evaluate the gradient diffusion strip method and disk diffusion methods to detect ceftazidime–avibactam susceptibility in China. The isolates collected in center 2 had distinct geographical characteristics; the 16 carbapenemase-positive isolates were all class B metallo-β-lactamases, and no *bla*_*KPC*_ positive isolate which had the highest isolation rate of carbapenemase resistance genes has been isolated (Zhang et al., 2018; Yang et al., 2020). A comparable number of *bla*_*KPC*_- and *bla*_*NDM*_-positive isolates which were the major carbapenemases concerned were investigated in this study (Zhang et al., 2017).

For *Enterobacterales*, no MEs and VMEs were found using both the gradient diffusion strip method and disk diffusion methods. In the 41 resistant *Enterobacteriaceae*, most of the MICs measured by BMD and gradient diffusion strip method showed a high level of resistance (MICs ≥ 256/4 mg/l), apart from one carbapenemase-non-producing isolate of *Serratia marcescens* (MIC = 32/4 mg/l). However, the disk zone diameter did not show high-level resistance, only two isolates’ inhibition zone diameters were <10 mm. Most disk zone diameters of resistant *Enterobacterales* (35 isolates) were distributed between 14 and 20 mm. Unlike the case of *Enterobacterales*, among the 14 resistant *P. aeruginosa* measured by the gradient diffusion strip method and BMD, the MICs were scattered between 16/4 and >256/4 mg/l. Eleven resistant *P. aeruginosa* isolates were detected by the disk diffusion method, and eight had an inhibition zone diameter between 17 and 20 mm. The remaining three isolates were resistant to BMD and susceptible to the disk diffusion method; their inhibition zone diameters were 21, 21, and 22 mm, respectively. All these three values are very close to 20 mm. The findings were consistent with those of a previous report when the disk diffusion method had more VME and was a universal result (Zhou et al., 2018; Wenzler et al., 2019; Wang et al., 2020). Most of carbapenemase-non-producing resistant *P. aeruginosa* isolates (11/14) exhibited low-level resistance (MIC was 16/4 or 32/4 mg/l), which is a point of concern. The mechanisms thought to have the greatest effect on *P. aeruginosa* compared to other Gram-negative microorganisms were the presence of inducible AmpC cephalosporinase expression, constitutive and inducible efflux pump production, and low outer membrane permeability (Dotsch et al., 2009; Chalhoub et al., 2018).

In the 31 *bla*_*KPC*_-positive *Enterobacterales* isolates, 30 were susceptible to ceftazidime–avibactam, in addition to *Klebsiella oxytoca* that *bla*_*KPC*_ coupled with *bla*_*NDM*_ positive. However, there have been some reports of *bla*_*KPC*_-positive *Klebsiella pneumoniae* resistance to ceftazidime–avibactam in recent years (Gottig et al., 2019; Antinori et al., 2020). In the 32 *bla*_*NDM*_-positive *Enterobacterales* isolates, only one *Escherichia coli* isolated from a rectal swab of a newborn was susceptible to ceftazidime–avibactam; the others were all resistant.

There was a particular limitation to our study. We were unable to isolate the *bla*_*OXA–*__48_-positive strains and sufficient *bla*_*VIM*_-positive strains. This was because these two carbapenemase types are very rare in China (Zhou et al., 2018; Wei et al., 2020). In future investigations, we will deliberately preserve both types of strains to facilitate future research.

## Conclusion

In conclusion, both the gradient diffusion strip method and the disk diffusion method met the needs of clinical microbiological laboratories for testing the susceptibility of ceftazidime–avibactam drugs. By comparison, the performance of the gradient strip was better than disk diffusion method. The detection performance of *Enterobacterales* was better than for *P. aeruginosa.*

## Data Availability Statement

The original contributions presented in the study are included in the article/Supplementary Material, further inquiries can be directed to the corresponding authors.

## Ethics Statement

Ethical review and approval was not required for the study on human participants, in accordance with the local legislation and institutional requirements.

## Author Contributions

HS, WJ, QY, and YX conceived and designed the experiments. JZ, GL, GZ, WK, SD, TW, JL, and ZH performed the experiments. JZ and GL analyzed the data. JZ wrote the manuscript. All authors approved the final version of the manuscript.

## Conflict of Interest

The authors declare that the research was conducted in the absence of any commercial or financial relationships that could be construed as a potential conflict of interest.

## Publisher’s Note

All claims expressed in this article are solely those of the authors and do not necessarily represent those of their affiliated organizations, or those of the publisher, the editors and the reviewers. Any product that may be evaluated in this article, or claim that may be made by its manufacturer, is not guaranteed or endorsed by the publisher.

## Footnotes

1
http://www.ncbi.nlm.nih.gov/BLAST
